# Triglyceride-glucose index at ICU admission predicts hospital mortality in patients with acute coronary syndrome concomitant sepsis: a Bayesian network analysis of retrospective multicenter cohort study

**DOI:** 10.3389/fnut.2026.1716973

**Published:** 2026-02-10

**Authors:** Yan Liu, Yingyi Luan, Lei Wang, Yinuo Zhu, Guoying Zheng, Jinxia Zhang, Zhifeng Liu, Yongming Yao, Ming Wu

**Affiliations:** 1 Department of Infection and Critical Care Medicine, Shenzhen Second People’s Hospital, Shenzhen, China; 2 Department of Nosocomial Infection Prevention and Control, Shenzhen Second People’s Hospital, Shenzhen, China; 3 Department of Central Laboratory, Beijing Obstetrics and Gynecology Hospital, Capital Medical University, Beijing, China; 4 The First School of Clinical Medicine, Southern Medical University, Guangzhou, China; 5 Department of Critical Care Medicine, Central People's Hospital of Zhanjiang, Zhanjiang, Guangdong, China; 6 Department of Medical Critical Care Medicine, General Hospital of Southern Theatre Command of PLA, Guangzhou, China; 7 Department of Critical Care Medicine, Huadu District People's Hospital of Guangzhou, Guangdong, China; 8 Department of Cardiovascular, General Hospital of Southern Theatre Command of PLA, Guangzhou, China; 9 Trauma Research Center, Medical Innovation Research Department and Fourth Medical Center of the Chinese PLA General Hospital, Beijing, China; 10 Department of Hematology, The First Affiliated Hospital of Shenzhen University, Shenzhen Second People’s Hospital, Shenzhen, China

**Keywords:** acute coronary syndrome, sepsis, triglyceride-glucose index, mortality, Bayesian network, critical care

## Abstract

**Background:**

Critically ill patients with acute coronary syndrome (ACS) concomitant sepsis are at markedly increased risk of mortality. The Triglyceride-Glucose (TyG) index, a simple surrogate marker of insulin resistance, although its predictive in separate cardiovascular or septic cohorts, its prognostic utility and potential mechanistic pathways in the high-risk setting of concurrent ACS concomitant sepsis remain unknown. This study aimed to evaluate the association between the TyG index at ICU admission and hospital mortality in this population and to elucidate underlying metabolic pathways using Bayesian network analysis.

**Methods:**

In a multicenter retrospective cohort of 200 critically ill adults with ACS concomitant sepsis (2013–2023), the TyG index was calculated at ICU admission and stratified into tertiles (T1: <8.81; T2: 8.81–9.45; T3: >9.45). The primary outcome was in-hospital mortality. Multivariable logistic regression and restricted cubic splines were used to assess the independent relationship between the TyG index and mortality. A Bayesian network (BN) model was constructed to infer causal interactions among metabolic variables, the TyG index and mortality.

**Results:**

Overall hospital mortality was 61.0% and increased significantly across TyG tertiles (T1: 54.5%; T2: 55.2%; T3: 73.1%; *p* = 0.044). After adjustment for confounders including age and peak procalcitonin, each unit increase in the TyG index was independently associated with higher mortality (adjusted odds ratio = 1.59; 95% confidence interval: 1.06–2.38; *p* = 0.026). A linear dose–response relationship was observed (*p* for nonlinearity = 0.549). The Bayesian network identified two primary metabolic pathways influencing TyG: Age→Triglycerides→TyG and Age→Diabetes→TyG. Importantly, a direct causal link from the TyG index to mortality (TyG → Mortality) was established. Setting the TyG index to its highest tertile alone predicted a mortality probability of 69.5%, with upstream metabolic factors providing minimal incremental prognostic value.

**Conclusion:**

In critically ill patients with ACS concomitant sepsis, a higher TyG index at ICU admission, reflecting insulin resistance and metabolic dysfunction, is a strong and independent predictor of hospital mortality. It occupies a central position linking age-related metabolic deterioration to fatal outcomes. Incorporation of the TyG index into early risk stratification may help identify patients who could benefit from intensified metabolic monitoring and tailored nutritional therapeutic strategies.

## Graphical abstract

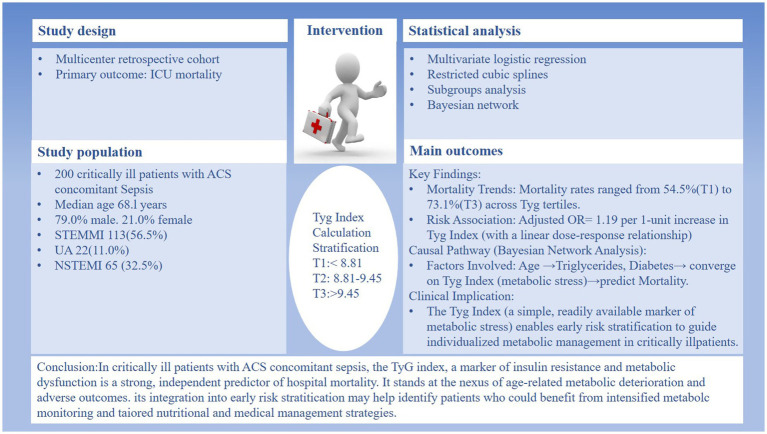

## Introduction

Acute coronary syndrome (ACS) and sepsis are two critical conditions that frequently lead to adverse outcomes. Both share overlapping pathophysiological mechanisms, including systemic inflammation, oxidative stress, and profound metabolic disturbances. When ACS and sepsis coexist, these synergistic processes amplify endothelial dysfunction and multiorgan injury, substantially increasing mortality risk (1–3). Identifying reliable prognostic biomarkers in this complex setting is therefore of great clinical relevance, as early risk stratification could allow timely, targeted interventions to improve outcomes.

Insulin resistance (IR) is a pivotal mechanism linking dyslipidemia, hyperglycemia, and inflammation in both cardiovascular and infectious diseases (4). The triglyceride–glucose (TyG) index, calculated from fasting triglyceride and glucose levels, has been validated as a simple and robust surrogate marker of IR (5–7). Elevated TyG index has been associated with adverse cardiovascular outcomes, including ACS and heart failure (1, 8–11) or with poor prognosis in sepsis (3, 12). It reflects the dual burden of impaired glucose homeostasis and dyslipidemia, especially hypertriglyceridemia, both of which play key roles in atherosclerotic progression and inflammatory activation. Moreover, insulin resistance itself has been directly implicated in worse outcomes for ACS patients. For example, in diabetic ACS patients, improving insulin sensitivity with an SGLT2 inhibitor (dapagliflozin) was reported to reduce microvascular complications (no-reflow phenomenon) and improve outcomes (13). Likewise, effective management of insulin resistance in ACS is recommended to enhance prognosis (14). In addition, the TyG index has recently been highlighted as a predictor of coronary artery disease complexity in elderly ACS patients (15), underscoring its clinical relevance as an IR marker. Despite these findings, the prognostic significance of the TyG index in patients with ACS complicated by sepsis remains uncertain. Given the convergence of metabolic and inflammatory derangements in this population, the TyG index may serve as an integrative biomarker of mortality risk.

Most prior studies have used conventional regression analyses, which quantify associations but are limited in uncovering complex, directional relationships among metabolic risk factors and outcomes. Bayesian network (BN) modeling provides a probabilistic framework that can visualize multistep pathways and potential causal interactions even in modestly sized datasets (16–18). Therefore, this multicenter retrospective study aimed to investigate the association between the TyG index and in-hospital mortality among patients with ACS complicated by sepsis. Furthermore, BN analysis was employed to explore potential mechanistic pathways linking metabolic abnormalities, TyG index, and adverse outcomes.

## Methods

### Study population

The retrospective multicentre cohort study screened consecutive adults admitted to the intensive care units of three tertiary (Class III Grade A) hospitals in southern China between January 2013 and December 2023. Patients were eligible if they met all of the following: (i) age ≥ 18 years; (ii) diagnosis of acute coronary syndrome (ACS) according to the 2023 European Society of Cardiology guidelines (19); and (iii) concomitant sepsis as defined by Sepsis-3 (suspected or confirmed infection an acute increase of≥2 points in the Sequential Organ Failure Assessment (SOFA) score from baseline) (20).

The exclusion criteria were as following: (i) pregnancy or lactation; (ii) long-term immunosuppression or severe immunodeficiency; (iii) terminal malignancy; (iv) irreversible disease or end-of-life status; (v) pre-existing infection predating the index ACS event; (vi) refusal of active treatment during hospitalization; and (vii) previous bone marrow or solid-organ transplantation.

### Data collection and definitions

On the basis of a reported 34.7% (21) hospital mortality in sepsis, at least 178 subjects were required to estimate this proportion with a 95% confidence interval half-width of 7%; because mortality in ACS complicated by sepsis is higher, our final sample of 200 patients provides adequate precision and events-per-variable for multivariable analysis. Clinical data, including demographics, comorbidities, laboratory results, and clinical outcomes, were retrieved from electronic medical records. The TyG index was calculated as ln [triglycerides (mg/dL) × fasting glucose (mg/dL)/2] (22–25). Patients were divided into tertiles based on the TyG index: T1 (<8.81), T2 (8.81–9.45), and T3 (>9.45).

### Statistical analysis

Continuous variables are presented as mean±standard deviation or median (interquartile range), depending on their distribution, and compared using Student’s *t*-test or one-way ANOVA for normally distributed data, and the Wilcoxon rank-sum test for non-normal data. Categorical variables are presented as counts (percentages) and were compared using the chi-square test or Fisher’s exact test, as appropriate. For partially lacking datasets in clinical characteristic data, we applied multiple imputation to fill in the missing values. To explore potential nonlinear relationships between TyG index levels and the mortality, a restricted cubic spline analysis was conducted. The relationship between TyG index and mortality was then assessed by multivariable logistic regression, with odds ratios (ORs) and 95% confidence intervals (CIs) estimated under three models: Model 1 (unadjusted, TyG index only), Model 2 (adjusted for age and gender), and Model 3 (adjusted for age, gender, coronary artery disease, and maximum procalcitonin). Covariates were selected *a priori* based on clinical relevance and variables with *p* < 0.1 in univariate logistic regression. Variance inflation factors (VIFs) were examined to detect multicollinearity among the variables. Furthermore, a subgroup analysis stratified by age (< 70 vs. ≥ 70 years), gender, infarction type (ST segment elevation myocardial infarction (STEMI), non-ST segment elevation myocardial infarction (NSTEMI), and unstable angina (UA)), and other key clinical factors was conducted to validate the association between TyG index and the mortality using logistic models adjusted for age, gender, coronary artery disease, and maximum procalcitonin. Interaction terms were tested by likelihood-ratio tests.

Subsequently, Bayesian network analysis was performed to create and evaluate plausible causal relationships between the TyG index and the mortality. The Bayesian network is a directed acyclic graph in which each node represents a random variable and the arcs linking the nodes represent relationships (conditional dependencies) between variables (26). The construction of a Bayesian network includes Bayesian learning and Bayesian inference. The Max-Min Hill Climbing (MMHC) algorithm was used for the structure learning (27).

All statistical analyses were performed using R version 4.4.2 (R Foundation). Statistical significance was defined as a two-sided *p*-value of <0.05.

## Results

### Baseline characteristics and outcomes

Of the 256 patients who satisfied these criteria, 56 were excluded because of substantial missing clinical data, leaving 200 participants for the final analysis. The inclusion and exclusion criteria resulted in the enrollment of 200 ACS patients with sepsis. Of the 200 patients analyzed, the median age was 68 years, and 79.0% were male. The distribution of ACS subtypes included 56.5% STEMI, 32.5% NSTEMI, and 11.0% UA (Table 1). Age, sex and ACS subtype were evenly distributed across TyG tertiles (*p* > 0.05). Admission haemodynamics were similar among groups; however, inflammatory and metabolic markers increased with TyG: white blood cell count (*p* = 0.010), neutrophil percentage (*p* = 0.002), platelet count (*p* = 0.016), fasting glucose and triglycerides (both *p* < 0.001), total cholesterol (*p* = 0.008) and sodium (*p* = 0.019); and mean corpuscular hemoglobin concentration decreased (*p* = 0.011).

**Table 1:** Baseline characteristics and outcomes of participants categorized by TyG index.

Characteristic and outcomes	Total (*N* = 200)	T1 (*N* = 66)	T2 (*N* = 67)	T3 (*N* = 67)	*p* value
Age(years) (mean (SD))	68.1 (13.6)	69.2 (14.1)	69.5 (12.1)	65.7 (14.4)	0.207
Gender, *n* (%)					
Male	158 (79.0)	54 (81.8)	54 (80.6)	50 (74.6)	0.551
Female	42 (21.0)	12 (18.2)	13 (19.4)	17 (25.4)	
ACS subtypes, *n* (%)					
STEMI	113 (56.5)	38 (57.6)	35 (52.2)	40 (59.7)	0.483
UA	22 (11.0)	4 (6.1)	10 (14.9)	8 (11.9)	
NSTEMI	65 (32.5)	24 (36.4)	22 (32.8)	19 (28.4)	
Insulin, *n* (%)	16 (8.0)	5 (7.6)	2 (3.0)	9 (13.4)	0.082
Oral hypoglycemic agents, *n* (%)	40 (20.0)	9 (13.6)	14 (20.9)	17 (25.4)	0.233
Smoking, *n* (%)	44 (22.0)	8 (12.1)	18 (26.9)	18 (26.9)	0.061
Hypertension, *n* (%)	97 (48.5)	25 (37.9)	38 (56.7)	34 (50.7)	0.085
Diabetes mellitus, *n* (%)	68 (34.0)	15 (22.7)	22 (32.8)	31 (46.3)	0.016
Gastric disease, *n* (%)	13 (6.5)	2 (3.0)	7 (10.4)	4 (6.0)	0.217
Congestive heart failure, *n* (%)	5 (2.5)	0 (0.0)	3 (4.5)	2 (3.0)	0.243
Chronic kidney disease, *n* (%)	22 (11.0)	10 (15.2)	7 (10.4)	5 (7.5)	0.361
Dialysis, *n* (%)	6 (3.0)	4 (6.1)	1 (1.5)	1 (1.5)	0.205
Stroke, *n* (%)	28 (14.0)	5 (7.6)	10 (14.9)	13 (19.4)	0.14
COPD, *n* (%)	16 (8.0)	8 (12.1)	6 (9.0)	2 (3.0)	0.143
Coronary artery disease, *n* (%)	33 (16.5)	12 (18.2)	12 (17.9)	9 (13.4)	0.708
Valvular heart disease, *n* (%)	5 (2.5)	1 (1.5)	2 (3.0)	2 (3.0)	0.822
Myocardial infarction, *n* (%)	17 (8.5)	8 (12.1)	5 (7.5)	4 (6.0)	0.415
Percutaneous coronary intervention, *n* (%)	25 (12.5)	9 (13.6)	8 (11.9)	8 (11.9)	0.944
Stent placement, *n* (%)	16 (8.0)	6 (9.1)	6 (9.0)	4 (6.0)	0.754
Systolic blood pressure (mmHg), mean (SD)	110.7 (26.2)	111.6 (29.3)	111.0 (26.1)	109.5 (23.3)	0.892
Diastolic blood pressure (mmHg), mean (SD)	63.8 (15.5)	62.8 (17.6)	65.1 (13.9)	63.5 (14.9)	0.689
Heart rate (bpm), mean (SD)	98.4 (23.0)	97.4 (22.0)	97.0 (21.4)	100.7 (25.4)	0.589
Temperature (°C), mean (SD)	37.0 (2.0)	36.8 (2.9)	37.1 (1.1)	37.2 (1.6)	0.584
Red blood cell count (1 × 10^12^/L), mean (SD)	3.8 (0.9)	3.7 (0.8)	3.8 (0.9)	4.0 (1.0)	0.207
Hemoglobin (g/L), mean (SD)	114.0 (25.9)	111.6 (27.1)	113.4 (25.5)	117.0 (25.1)	0.47
Hematocrit (%), median [IQR]	0.4 [0.3, 0.4]	0.3 [0.3, 0.4]	0.4 [0.3, 0.4]	0.4 [0.3, 0.4]	0.495
MCHC (g/dL), mean (SD)	329.2 (14.9)	333.6 (15.1)	328.1 (13.0)	326.1 (15.6)	0.011
WBC (1 × 10^9^/L), median [IQR]	15.9 [11.9, 19.9]	13.8 [10.5, 18.5]	15.6 [11.1, 19.8]	17.4 [14.1, 22.7]	0.01
Neutrophil (1 × 10^9^/L), median [IQR]	13.5 [9.8, 17.5]	11.9 [8.2, 14.9]	13.6 [9.7, 17.6]	15.0 [12.2, 19.4]	0.002
Lymphocyte (1 × 10^9^/L), median [IQR]	1.0 [0.6, 1.5]	1.0 [0.6, 1.6]	1.0 [0.7, 1.4]	1.0 [0.6, 1.6]	0.88
Monocyte (1 × 10^9^/L), median [IQR]	0.8 [0.6, 1.2]	0.8 [0.6, 1.1]	0.8 [0.6, 1.1]	0.9 [0.5, 1.3]	0.365
Platelet (1 × 10^9^/μL), median [IQR]	162.0 [120.8, 244.8]	138.0 [104.8, 204.5]	160.0 [116.0, 270.5]	188.0 [143.0, 262.5]	0.016
APTT (s), median [IQR]	45.8 [39.2, 62.0]	46.3 [42.5, 65.5]	44.3 [38.9, 54.3]	45.5 [38.0, 60.8]	0.289
D-Dimer (μg/mL), median [IQR]	3.4 [1.4, 7.3]	3.8 [1.4, 9.5]	3.7 [1.5, 7.0]	2.7 [1.5, 6.2]	0.802
INR, median [IQR]	1.3 [1.1, 1.6]	1.3 [1.2, 1.7]	1.3 [1.1, 1.7]	1.2 [1.1, 1.5]	0.606
Fibrinogen (g/L), median [IQR]	4.9 [3.3, 6.4]	4.7 [3.0, 6.3]	4.7 [3.4, 6.4]	5.2 [3.6, 6.6]	0.44
Procalcitonin (ng/ml), median [IQR]	3.5 [0.7, 14.2]	5.2 [1.0, 18.3]	2.4 [0.4, 12.7]	3.6 [0.9, 12.5]	0.248
Maximum procalcitonin (ng/ml), median [IQR]	11.6 [3.2, 28.7]	13.2 [2.9, 33.8]	8.8 [1.8, 29.4]	12.0 [4.9, 26.2]	0.597
CK-MB (U/L), median [IQR]	32.0 [10.0, 101.0]	46.2 [14.1, 111.0]	39.4 [10.1, 118.2]	26.9 [6.0, 76.7]	0.296
Albumin (g/L), mean (SD)	32.7 (5.3)	32.1 (4.7)	32.6 (5.4)	33.2 (5.8)	0.469
Creatinine (μmol/L), median [IQR]	177.5 [121.0, 271.0]	174.0 [120.2, 371.2]	173.0 [115.5, 259.0]	180.0 [127.0, 257.0]	0.494
Blood glucose (mmol/L), median [IQR]	9.6 [7.6, 14.1]	7.9 [6.6, 9.1]	9.8 [7.9, 11.9]	14.8 [9.9, 16.9]	<0.001
Total cholesterol (mmol/L), median [IQR]	4.1 [3.3, 5.0]	3.8 [2.9, 4.6]	4.1 [3.4, 4.9]	4.6 [3.5, 5.4]	0.008
Triglycerides (mmol/L), median [IQR]	1.2 [0.8, 1.7]	0.7 [0.6, 0.9]	1.2 [1.0, 1.5]	1.9 [1.5, 2.5]	<0.001
Total bilirubin (μmol/L), median [IQR]	14.6 [9.4, 24.6]	15.8 [9.7, 24.3]	15.4 [9.8, 26.6]	14.1 [8.9, 21.6]	0.392
Uric acid (μmol/L), median [IQR]	448.0 [299.8, 600.0]	393.0 [242.2, 512.5]	471.1 [314.5, 628.5]	480.0 [339.5, 620.0]	0.064
Alanine aminotransferase (U/L), median [IQR]	96.7 [36.8, 479.2]	100.0 [35.8, 1130.1]	115.0 [45.5, 314.0]	76.0 [30.5, 417.5]	0.411
Aspartate aminotransferase (U/L), median [IQR]	218.0 [77.0, 811.2]	168.0 [73.0, 1261.5]	238.0 [84.2, 720.5]	229.0 [62.0, 755.7]	0.851
Creatine kinase (U/L), median [IQR]	1315.7 [351.2, 4158.8]	1358.2 [410.4, 4653.8]	1314.0 [267.0, 3993.2]	1200.0 [371.1, 3303.0]	0.856
PaO_2_ (mmHg), median [IQR]	98.4 [80.2, 129.8]	98.8 [77.3, 130.3]	102.6 [82.9, 131.2]	95.3 [81.9, 126.9]	0.718
PaCO_2_ (mmHg), median [IQR]	35.8 [30.7, 43.1]	35.4 [30.6, 45.0]	36.0 [31.6, 42.5]	35.5 [25.9, 42.7]	0.529
Oxygen saturation (%), mean (SD)	95.3 (8.6)	94.9 (6.5)	94.8 (12.5)	96.1 (4.8)	0.604
Anion gap (mEq/L), median [IQR]	14.5 [10.5, 19.1]	14.0 [10.5, 18.6]	14.7 [10.2, 19.2]	15.3 [11.1, 19.4]	0.675
Potassium (mmol/L), mean (SD)	4.2 (0.7)	4.3 (0.6)	4.2 (0.8)	4.2 (0.8)	0.835
Sodium (mmol/L), median [IQR]	141.0 [137.9, 146.0]	139.1 [136.7, 143.0]	141.3 [138.0, 146.5]	142.0 [139.0, 148.0]	0.019
Chloride (mmol/L), mean (SD)	103.7 (11.2)	101.1 (14.7)	104.7 (8.7)	105.3 (9.0)	0.061
Phosphate (mg/dL), median [IQR]	1.2 [0.9, 1.7]	1.2 [0.8, 1.5]	1.3 [0.9, 1.9]	1.4 [1.0, 1.9]	0.143
Lactate (mmol/L), median [IQR]	3.1 [2.0, 6.0]	3.1 [2.2, 5.4]	2.6 [1.8, 7.4]	3.5 [2.2, 6.1]	0.684
Intensive care unit time (days), median [IQR]	1.0 [0.0, 3.0]	1.0 [0.0, 2.0]	1.0 [0.0, 4.0]	1.0 [0.0, 3.0]	0.423
Hospitalization time (days), median [IQR]	13.0 [7.0, 21.2]	12.5 [6.0, 21.0]	15.0 [6.5, 23.0]	14.0 [9.5, 21.0]	0.712
Hospital mortality, *n* (%)	122 (61.0)	36 (54.5)	37 (55.2)	49 (73.1)	0.044

ACS, acute coronary syndrome; STEMI, ST segment elevation myocardial infarction; NSTEMI, non-ST segment elevation myocardial infarction; UA, unstable angina; COPD, chronic obstructive pulmonary disease; bpm, beats per minute; MCHC, mean corpuscular hemoglobin concentration; WBC, white blood cell count; APTT, activated partial thromboplastin time; INR, international normalized ratio; CK-MB, Creatine kinase isoenzymes; PaO_2_, arterial partial pressure of oxygen; PaCO_2_, arterial partial pressure of carbon dioxide; TyG index, triglyceride-glucose index.

As shown in Table 1, ICU stay (median 1 day, IQR 0–3) and overall hospital stay (median 13 days, IQR 7–21) did not differ by tertiles (*p* = 0.423 and 0.712). However, the mortality increased significantly with rising TyG levels: 54.5% in T1, 55.2% in T2, and 73.1% in T3 (*p* = 0.044).

### Association of TyG index and the mortality

**Figure 1:**
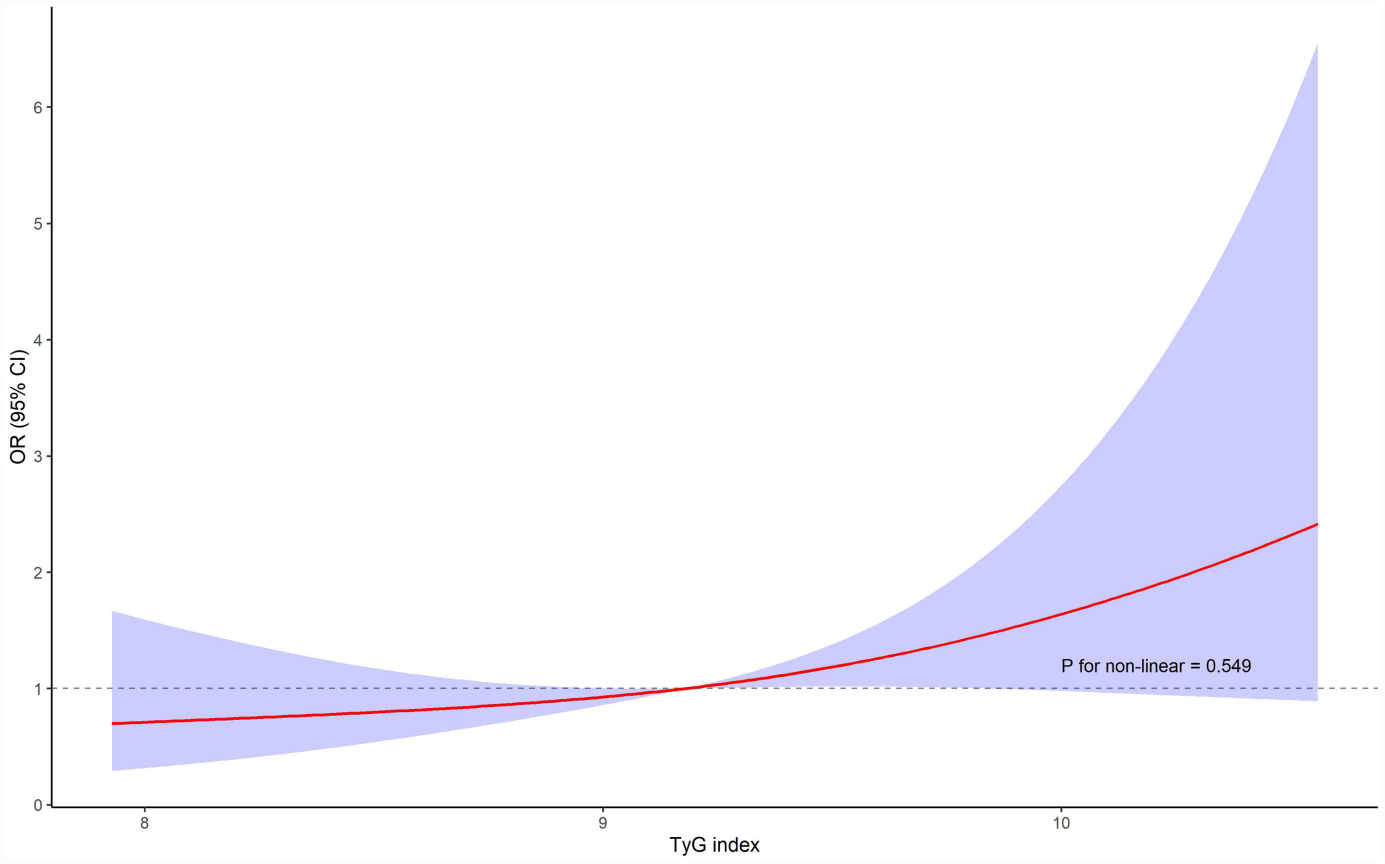
Restricted cubic spline curve for the association between the TyG index and the risk of the mortality. Red lines represent odds ratios, and blue areas represent 95% confidence intervals. The model was adjusted for age, gender, coronary artery disease, maximum procalcitonin.

Multivariate logistic regression analysis showed that the TyG index was associated with an elevated risk of the mortality (OR 1.59[95% CI 1.06–2.38]; *p* = 0.026). Compared with the lowest TyG tertile (T1), the highest tertile (T3) showed a significantly increased risk of mortality in all models (Model 3: OR 2.54, 95% CI: 1.16–5.56, *p* = 0.020). There was a significant linear trend across tertiles (*p* for trend = 0.021), further highlighting the dose–response relationship (Table 2). Restricted cubic spline analysis confirmed a linear relationship between the TyG index and mortality risk, with no threshold or plateau effect observed (p for nonlinearity = 0.549, Figure 1).

**Table 2:** ORs (95% CIs) for the mortality according to the TyG index.

Variable	Model 1		Model 2		Model 3	
	OR (95% CI)	*p*-value	OR (95% CI)	*p*-value	OR (95% CI)	*p*-value
TyG index	1.52 (1.04–2.21)	0.031	1.54 (1.05–2.25)	0.028	1.59 (1.06–2.38)	0.026
TyG index tertile						
T1	ref		ref		ref	
T2	1.03 (0.52–2.04)	0.937	1.02 (0.51–2.03)	0.956	1.08 (0.52–2.24)	0.847
T3	2.27 (1.10–4.69)	0.027	2.27 (1.09–4.75)	0.029	2.54 (1.16–5.56)	0.02
*p* for trend		0.029		0.032		0.021

Model 1: Unadjusted.

Model 2: Adjusted for age, gender.

Model 3: Adjusted for age, gender, Coronary Artery Disease, Maximum procalcitonin.

OR, odds ratio; CI, confidence interval; TyG index, triglyceride-glucose index.

### Subgroup analysis

**Figure 2:**
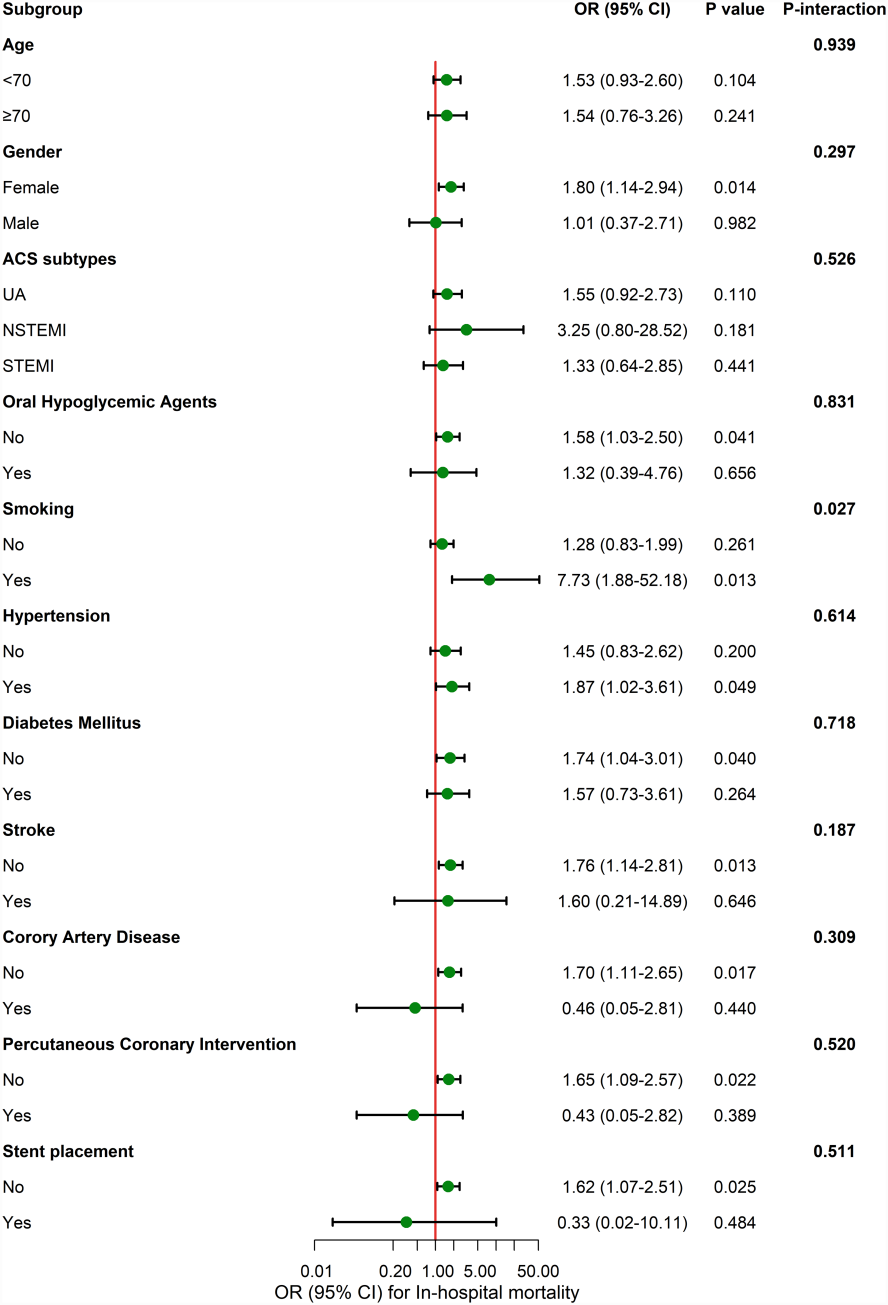
Subgroup analyses for the association of TyG index with the mortality. Adjusted for age, gender, coronary artery disease, and maximum procalcitonin. OR, Odds ratio; CI, Confidence interval.

In subgroup analyses adjusted for age, gender, coronary artery disease and maximum procalcitonin, the association between TyG index and mortality was broadly consistent across almost all clinical strata and there was no statistically significant effect modification. The only exception was a minor interaction by smoking status (*p* for interaction = 0.027). The results of the stratified analysis consistently demonstrated a similar association of TyG index values across most sub-populations, as shown in Figure 2.

### Reciprocal causal inference by Bayesian network

**Figure 3:**
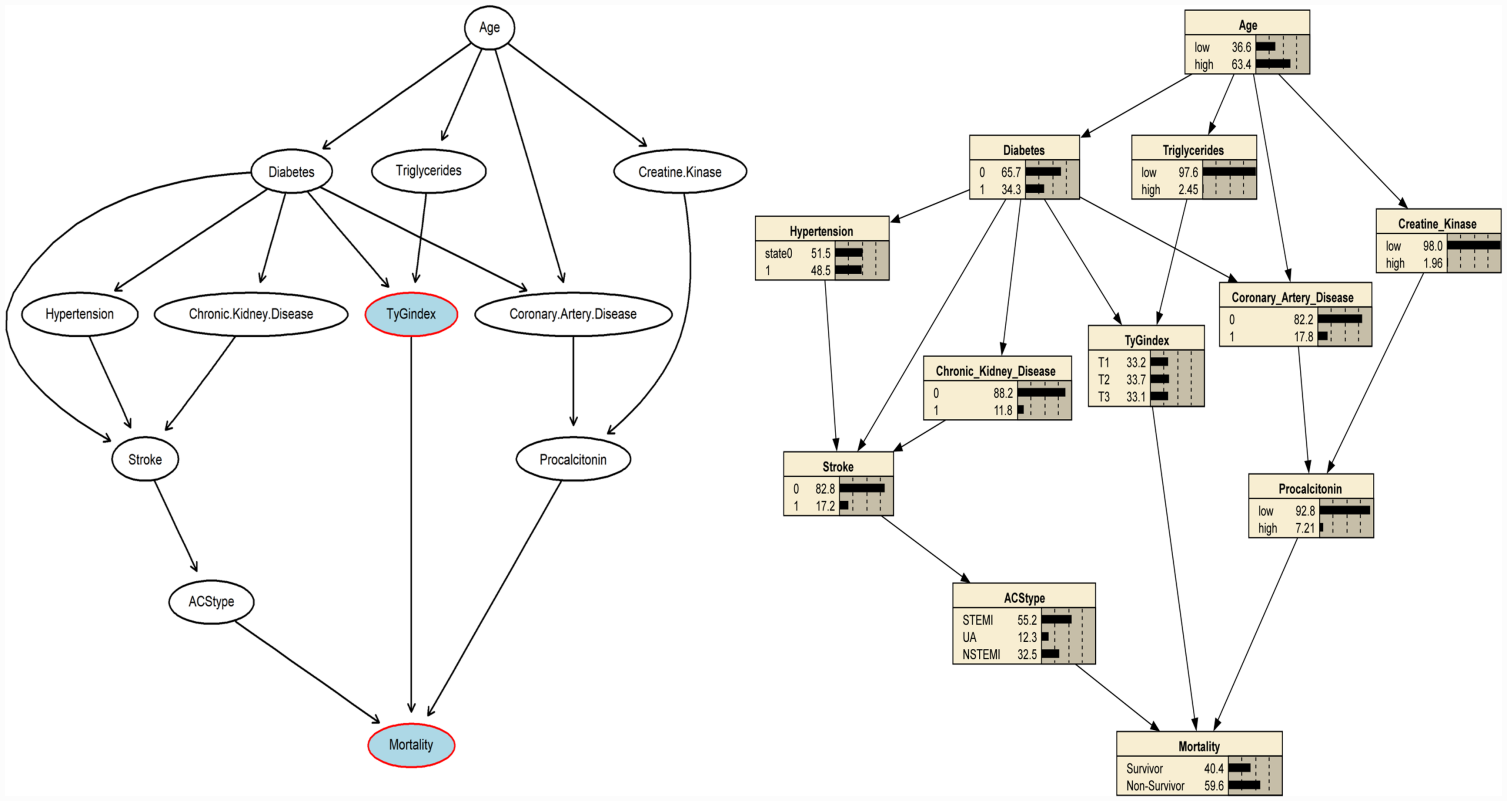
Bayesian network and conditional probabilities for the pathways to mortality.

After excluding nodes that were not related to the TyG index or mortality, a final Bayesian network with 12 nodes and 8 arcs was constructed (Figure 3). Two pathways were associated with elevated TyG index: a metabolic pathway in which advancing age increased fasting triglycerides that, in turn, raised TyG (Age→ Triglycerides→TyG), and a comorbidity pathway whereby age promoted diabetes mellitus, which likewise fed into TyG (Age→Diabetes→TyG). One pathway from TyG index to the mortality (TyG → Mortality).

**Figure 4:**
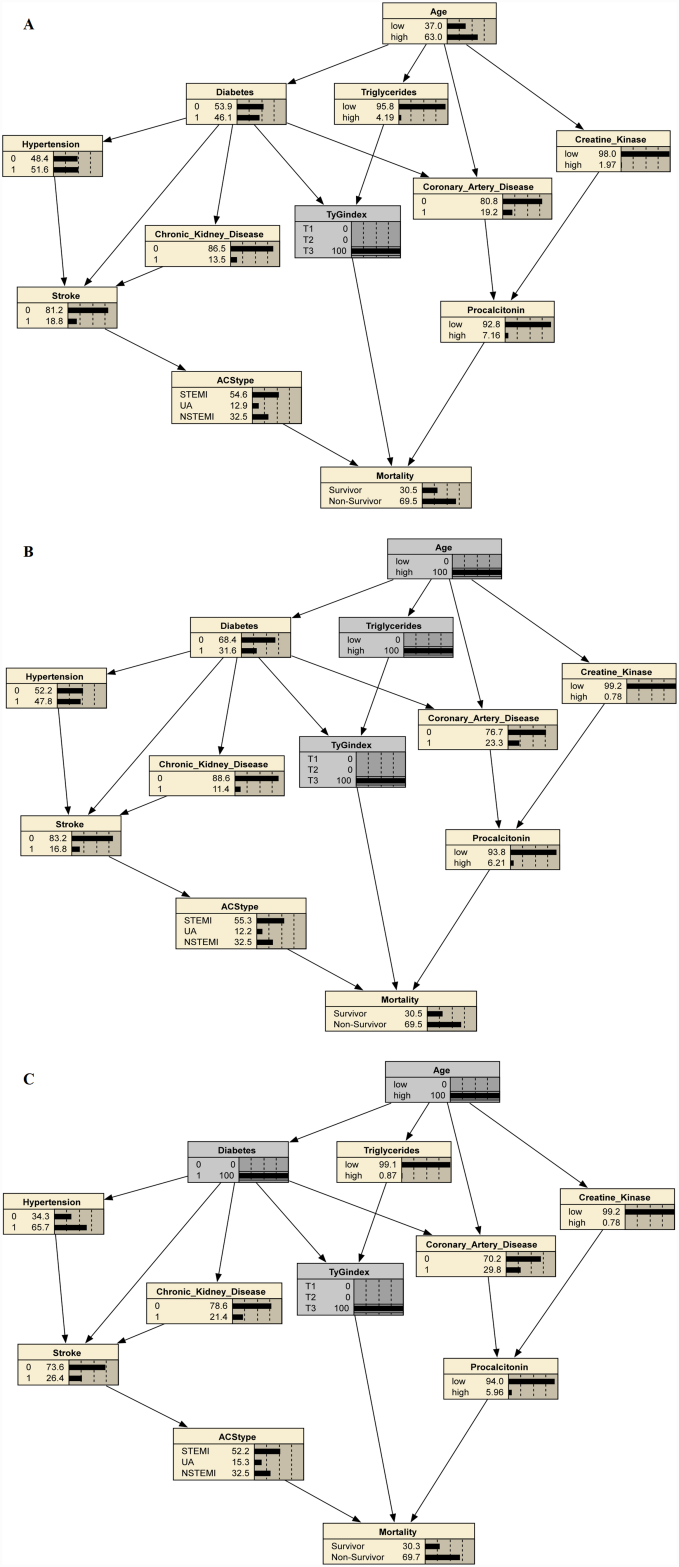
The maximum conditional probability for the pathways through elevated TyG index to mortality. **(A)** Predicted mortality probability (69.5%) when TyG index is set to the highest tertile (T3) alone. **(B)** Predicted mortality probability (69.5%) when the metabolic pathway (Age = high + Triglycerides = high → TyG = T3) is imposed. **(C)** Predicted mortality probability (69.7%) when the comorbidity pathway (Age = high + Diabetes = yes → TyG = T3) is imposed.

In the Bayesian network, elevating the TyG index to its highest tertile alone predicted an in-hospital mortality probability of 69.5% (Figure 4A); superimposing the metabolic pathway (Age = high + Triglycerides = high →TyG) produced an identical estimate of 69.5% (Figure 4B); and imposing the comorbidity pathway (Age = high + Diabetes = yes →TyG) yielded a comparable probability of 69.7% (Figure 4C). These two TyG-centered routes represented the highest-risk trajectories among the eight Bayesian pathways leading to death (Supplementary Table S1). Once TyG is elevated, upstream determinants therefore add minimal prognostic information, underscoring TyG as the dominant driver of mortality risk in this cohort.

## Discussion

This study provides three key findings in a multicenter cohort of critically ill patients with ACS complicated by sepsis. First, the TyG index, a simple surrogate of lipid–glucose metabolism, was independently and linearly associated with higher hospital mortality, even after extensive adjustment for demographic, cardiovascular, and inflammatory factors. Second, Bayesian network analysis revealed two upstream pathways (Age→Triglycerides→TyG and Age→Diabetes→TyG) contributing to TyG elevation and identified a direct link from TyG to mortality, highlighting its role as a dominant metabolic driver of adverse outcomes. Third, the TyG index showed a stronger correlation with inflammatory markers in the highest tertile, positioning it at the interface of metabolic and inflammatory dysregulation.

The overall mortality rate of 61.0% underscores the extreme vulnerability of patients at the intersection of acute coronary ischemia and systemic sepsis. This finding is consistent with the recognized synergy whereby sepsis amplifies metabolic dysfunction and inflammatory cascades, thereby aggravating cardiovascular injury (28–30). Notably, in our cohort, ICU and hospital lengths of stay were similar across TyG tertiles, whereas mortality rates varied significantly. This suggests that a higher TyG index identifies patients at increased risk, independent of hospitalization duration or severity. Our study extends prior evidence that established the prognostic value of the TyG index in isolated ACS (1, 8–11) and sepsis populations (3, 12). To our knowledge, this is the first study to evaluate its predictive value in patients with ACS concomitant sepsis, a subgroup characterized by overlapping metabolic derangements and inflammatory responses.

The novel application of Bayesian network analysis in this context offers a mechanistic hypothesis beyond traditional statistics. Bayesian network analysis provides mechanistic insights into how an elevated TyG index may confer a higher mortality risk, reinforcing the concept of a metabolic–inflammatory–vascular axis. Advancing age increases the TyG index through two parallel routes: one mediated by elevated triglycerides and the other by diabetes mellitus, after which TyG is directly linked to death, suggesting a plausible causal chain. Mechanistically, chronic insulin resistance can impair endothelial nitric oxide synthase activity, reducing nitric oxide–mediated vasodilation and precipitating micro- and macrovascular dysfunction (31–36). It also drives adverse microvascular remodeling, including capillary rarefaction and inadequate collateralization, through down-regulation of vascular endothelial growth factor signaling (33, 35, 37). Moreover, insulin resistance amplifies systemic inflammation; elevated interleukin-6 disrupts nitric oxide-mediated relaxation and intensifies vasoconstriction, further compromising myocardial perfusion (38–40). These intertwined pathways align with the direct TyG → mortality arc in our Bayesian model and plausibly explain the strong linear TyG index-mortality relationship observed in ACS complicated by sepsis.

The observed association between elevated TyG levels and increased inflammatory markers (for example, patients in the highest TyG tertile had significantly higher WBC counts and neutrophil percentages) further supports the concept of immunometabolic dysregulation. Insulin resistance promotes systemic inflammation via endothelial dysfunction, oxidative stress, and cytokine activation (e.g., interleukin-6), while inflammation can exacerbate insulin resistance and lipid imbalance (41–43). This bidirectional interaction may help explain the high mortality observed and aligns with the proposed metabolic–inflammatory–vascular axis (44).

Our Bayesian network model revealed a clinically significant pattern: when the TyG index reached its highest tertile, it became the dominant predictor of the mortality. Notably, incorporating upstream metabolic factors such as advanced age combined with hypertriglyceridemia, or advanced age with pre-existing diabetes, did not substantially alter the mortality probability beyond that predicted by the elevated TyG index alone. This robust finding underscores that once insulin resistance (as quantified by the TyG index) is established, it becomes the primary driver of mortality risk in ACS patients complicated by sepsis, with its antecedent conditions adding minimal incremental prognostic value.

Clinically, the TyG index offers a practical advantage as it can be derived from routine, inexpensive laboratory measurements and may serve as an adjunct for early risk stratification in ICU patients with ACS and sepsis. Identifying patients with elevated TyG may allow clinicians to intensify monitoring and consider tailored interventions. Although causality cannot be inferred, our findings raise the hypothesis that interventions aimed at improving insulin sensitivity and lipid metabolism (such as optimized glycemic control, omega-3 fatty acids, or insulin sensitizers (45–47)) could improve outcomes. Prospective multicenter studies with serial TyG measurements are needed to validate its prognostic value, assess its dynamic changes, and explore the impact of targeted therapies in this vulnerable population.

### Strengths and limitations

This study applied two methodological advantages that strengthened the validity of its conclusions. First, restricted cubic spline modeling was used to characterize the full exposure–response curve between the TyG index and mortality, avoiding arbitrary cut-points and revealing a linear relationship across the entire TyG range. Second, Bayesian-network analysis provided a transparent framework for exploring directional pathways, integrating metabolic, inflammatory and clinical variables, thus generating mechanistic hypotheses beyond conventional regression.

Furthermore, our sample size was carefully justified and represents a notable strength of this study. Based on a reported 34.7% hospital mortality rate in general sepsis populations, a minimum sample of 178 patients was required to estimate this proportion. Given that mortality is substantially higher in ACS complicated by sepsis, our final enrollment of 200 patients provided adequate statistical precision and sufficient events-per-variable for robust multivariable analyses. Importantly, this cohort is particularly valuable due to the inherent rarity and clinical complexity associated with ACS complicated by sepsis.

Our study has certain limitations. First, the TyG index was measured only at the time of sepsis diagnosis, and we did not capture dynamic changes in glucose and triglyceride levels during hospitalization, which might have prognostic significance. Although our use of Bayesian network analysis offers hypothesis-generating insights into possible causal pathways. Further prospective studies, ideally multi-center and including serial TyG measurements, are needed to confirm and expand our findings. Second, it would be worthwhile for future research to investigate whether interventions aimed at reducing insulin resistance and lowering TyG (such as optimal glycemic control and aggressive lipid management) can improve clinical outcomes in this vulnerable population.

## Conclusion

In patients with acute coronary syndrome complicated by sepsis, a higher triglyceride–glucose (TyG) index was independently and linearly associated with increased in-hospital mortality. The TyG index also tracked a heightened inflammatory milieu and, supported by Bayesian network analysis, emerged as a practical adjunct marker for early risk stratification based on routine laboratory tests. Incorporating TyG into prognostic assessment may help identify patients who warrant intensified monitoring and targeted metabolic management; prospective multicenter studies with serial TyG measurements are needed to confirm clinical utility and inform intervention strategies.

## Data Availability

The raw data supporting the conclusions of this article will be made available by the authors, without undue reservation.
