# Supplementary material for: Triglyceride-glucose index at ICU admission predicts hospital mortality in patients with acute coronary syndrome concomitant sepsis: a Bayesian network analysis of retrospective multicenter cohort study
Source: Front Nutr. 2026 Feb 10;13:1716973. doi: 10.3389/fnut.2026.1716973 (PMC12929510; doi:10.3389/fnut.2026.1716973)
Supplement: Supplementary file 1 [file Table_1.docx]

Table S1. The conditional probabilities of motality through different pathways

| Pathways to mortality | Condition probability, % | |
| --- | --- | --- |
| Age→Diabetes→TyG index→mortality | | 69.7 |
| Age→Triglycerides→TyG index→mortality | | 69.5 |
| Age→Diabetes→Stroke→ACS type→mortality | | 67.0 |
| Age→Diabetes→Chronic kidney Disease→Stroke→ACS type→mortality | | 67.0 |
| Age→Diabetes→Chronic kidney Disease→Stroke→ACS type→mortality | | 67.0 |
| Age→Diabetes→Hypertension→Stroke→ACS type→mortality | | 67.0 |
| Age→Diabetes→Coronary Artery Disease→Procalcitonin→mortality | | 60.3 |
| Age→Coronary Artery Disease→Procalcitonin→mortality | | 59.4 |
| Age→Creatine kinase→Procalcitonin→mortality | | 59.2 |
